# Supplementary material for: The EMT activator ZEB1 accelerates endosomal trafficking to establish a polarity axis in lung adenocarcinoma cells
Source: Nat Commun. 2021 Nov 3;12:6354. doi: 10.1038/s41467-021-26677-y (PMC8566461; doi:10.1038/s41467-021-26677-y)
Supplement: Supplementary file 1 — Supplementary Information [file 41467_2021_26677_MOESM1_ESM.pdf]

EMT activator ZEB1 accelerates endosomal trafficking to establish a polarity axis in lung adenocarcinoma cells

Supplementary Information

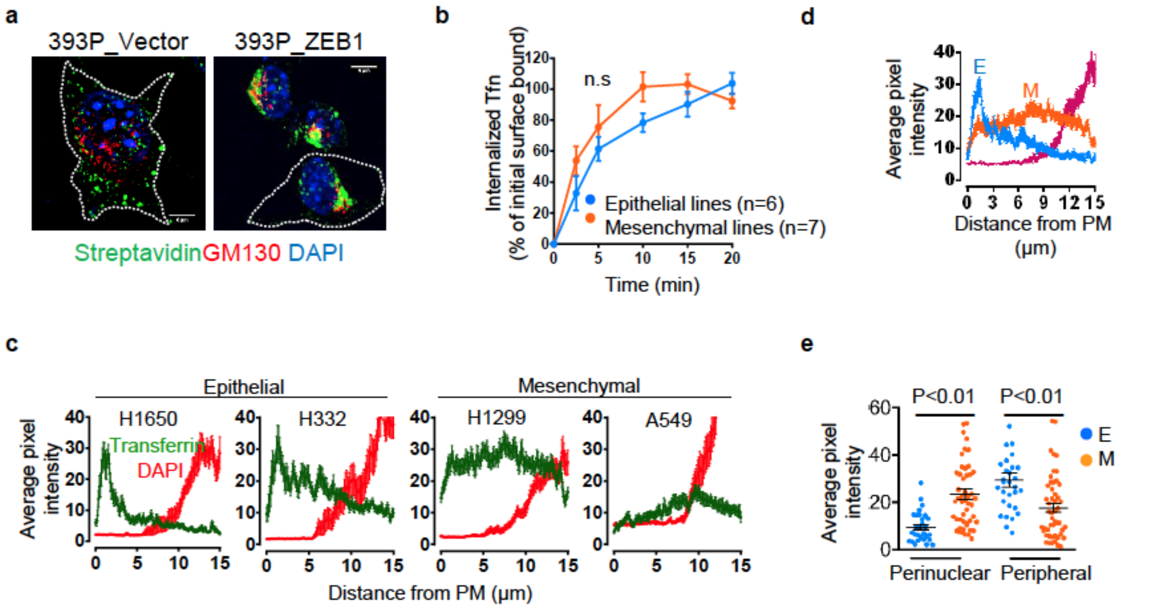

Supplementary Figure 1. Distinct transferrin (Tfn) trafficking patterns in epithelial and mesenchymal LUAD cells. (**a**) Merged confocal micrographs taken 30 min after initiating endocytosis of labeled biotin. Cells treated with Alexa-488 labelled streptavidin and stained with anti-GM130 to detect endocytosed biotin and Golgi, respectively. Cells are outlined (dashed lines). Scale bars: 5 µm. (**b**) In-cell ELISA of intracellular Tfn levels in epithelial (E, n=6) and mesenchymal (M, n=7) murine LUAD cell lines. Results normalized based on total protein content. (**c**) Alexa 568-labelled Tfn and DAPI intensities (Y axis) plotted on lines drawn from PM inwards (X axis) in human LUAD cell lines fixed 30 min after initiating endocytosis. Results are averages of 3 linescans per cell, ≥ 25 cells per cell line. (**d**) Tfn and DAPI signals from (**c**) were expressed as mean values for epithelial ('E', n=2) and mesenchymal ('M', n=2) human LUAD cell lines. (**e**) Tfn signal intensities in perinuclear and peripheral compartments of each cell (dot) from (**c**). n = 30 cells (epithelial) or 50 cells (mesenchymal) from 3 independent experiments. Data are presented as mean values +/- SEM; *P* values, two-tailed Student's t-test.

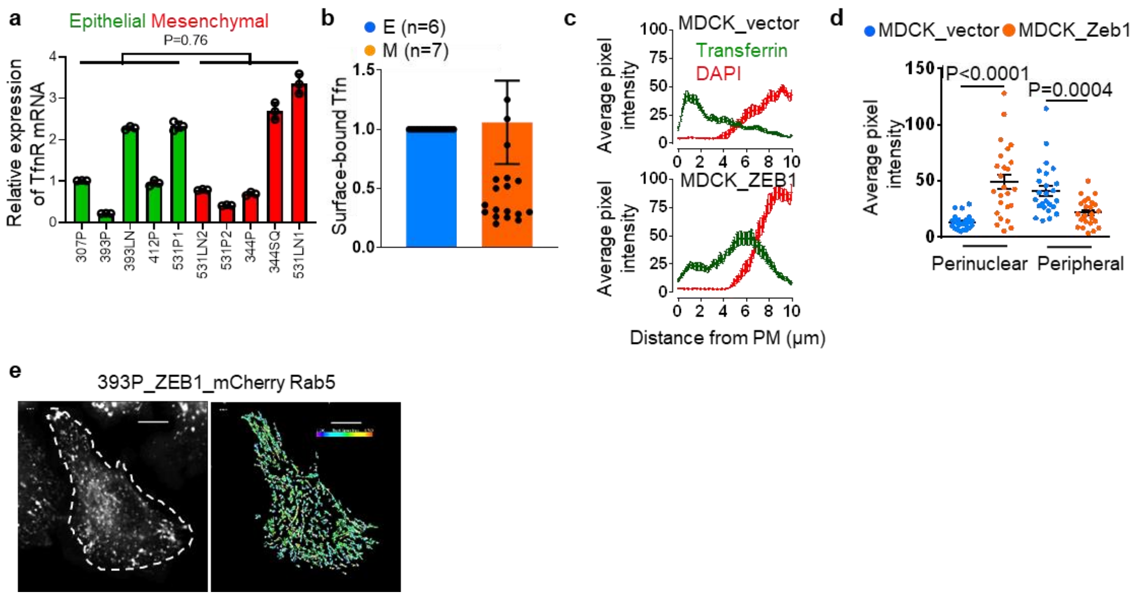

Supplementary Figure 2. ZEB1 accelerates endocytic vesicle trafficking. (**a**) qPCR analysis of transferrin receptor (TfnR) levels in murine LUAD cell lines. Values expressed relative to 307P. n = 3 independent experiments. (**b**) In-cell ELISA of surface-bound Tfn levels in epithelial ('E', n=6) and mesenchymal ('M', n=7) murine LUAD cell lines. Results normalized based on total protein content. (**c**) Alexa 568-labelled Tfn and DAPI signal intensities (Y axis) on lines drawn from PM inwards (X axis) in MDCK cells that have ectopic expression of ZEB1 or empty vector and were fixed 10 min after initiating endocytosis. Results are averages of 3 linescans per cell, n = 25 cells per cell line. (**d**) Tfn signal intensities in perinuclear and peripheral compartments of each cell (dot) from (**c**). Values represent the maximal signal intensities in each cell. n = 25 cells from 3 independent experiments. (**e**) Spinning disc confocal micrograph of a living 393P_ZEB1 cell stably expressing mCherry-tagged Rab5, an early endosomal marker (left image). Cells is outlined (dotted line). Vesicle tracks identified by automated vesicle tracking (right image). Live cell imaging was performed for 10 min at 1 sec intervals. Vesicle speed is color-coded (slow, blue; fast, red). Scale bars, 10 μm. Data are presented as mean values +/- SEM, or as mean values +/- SD (a); *P* values, two-tailed Student's t-test.

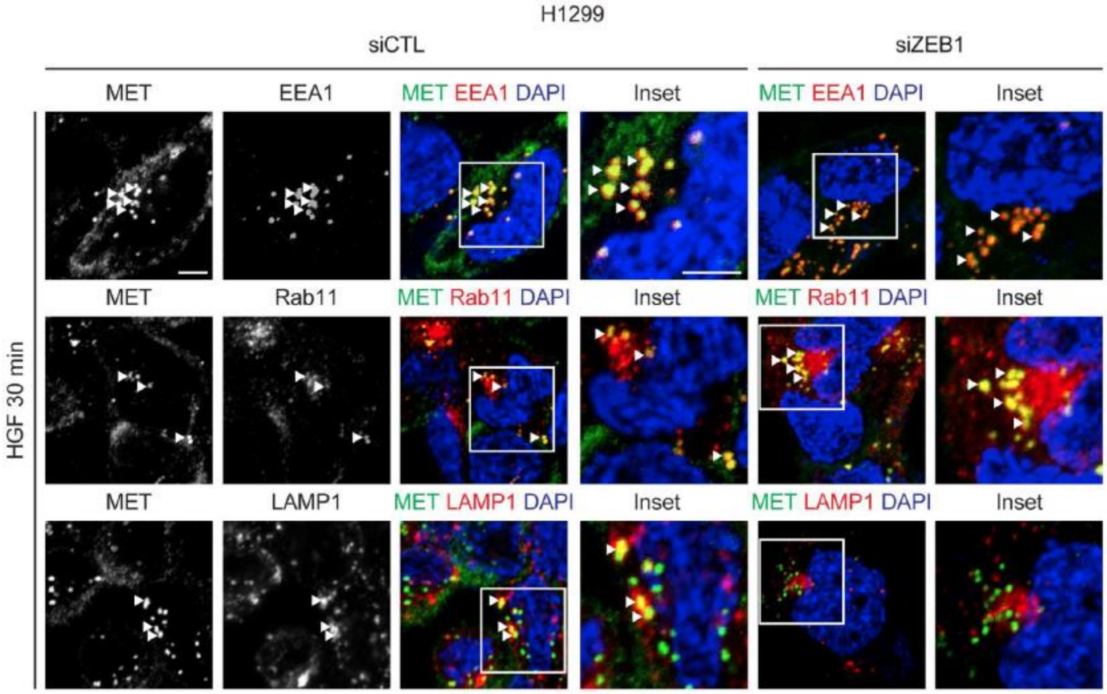

Supplementary Figure 3. ZEB1 influences intracellular trafficking of MET. Confocal images of control (siCTL) and ZEB1 siRNA-transfected H1299 cells co-stained with antibodies against endogenous MET, EEA1, Rab11, and LAMP1. Cells were incubated at 4°C with 250 ng/ml HGF for 30 min, washed, incubated at 37°C for 30 min, fixed, and stained. Single-channel and merged images (left-to-right, panels 1-2 and 3-6, respectively). Boxed areas are magnified (insets). Scale bars, 5 µm. Co-localized structures in siCTL-transfected cells are indicated (arrowheads). Quantified results are shown in Fig. 5b.

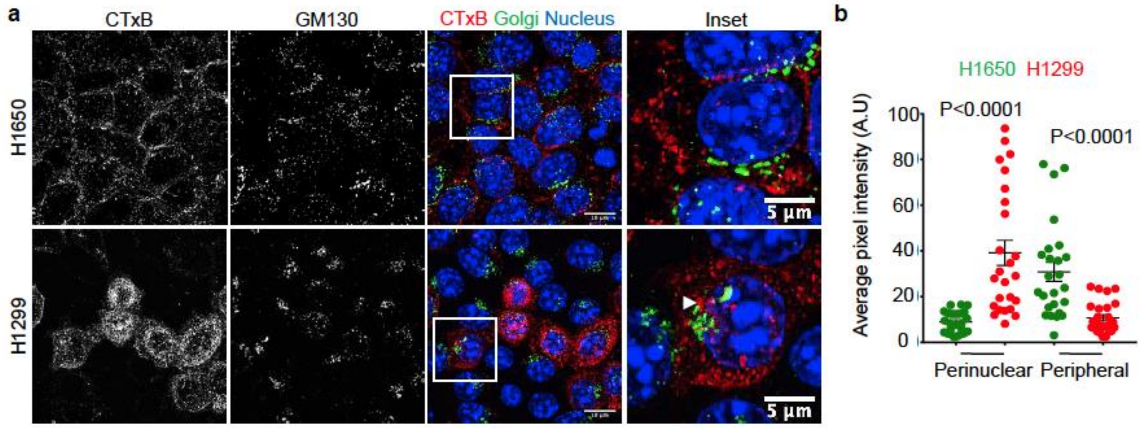

Supplementary Figure 4. Distinct cholera toxin B (CTxB) trafficking patterns in epithelial and mesenchymal human LUAD cells. (**a**) Single-channel and merged confocal micrographs taken 1 h after initiating Alexa 488-labeled CTxB treatment. Single-channel images show that CTxB staining is primarily PM-adjacent in epithelial H1650 cells and cytoplasmic in mesenchymal H1299 cells. CTxB co-localization with Golgi is detectable in H1299 cells, albeit far less so than at the same time point in 393P_ZEB1 cells (Fig. 6b) owing to a slower trafficking rate in the human cells. Scale bars: 10 µm, 5 µm (inset). (**b**) CTxB signal intensities in perinuclear and peripheral compartments of each cell (dot). n = 25 cells from 3 independent experiments. Values represent the maximal signal intensities in each cell. Data are presented as mean values +/- SEM; *P* values, two-tailed Student's t-test.

.

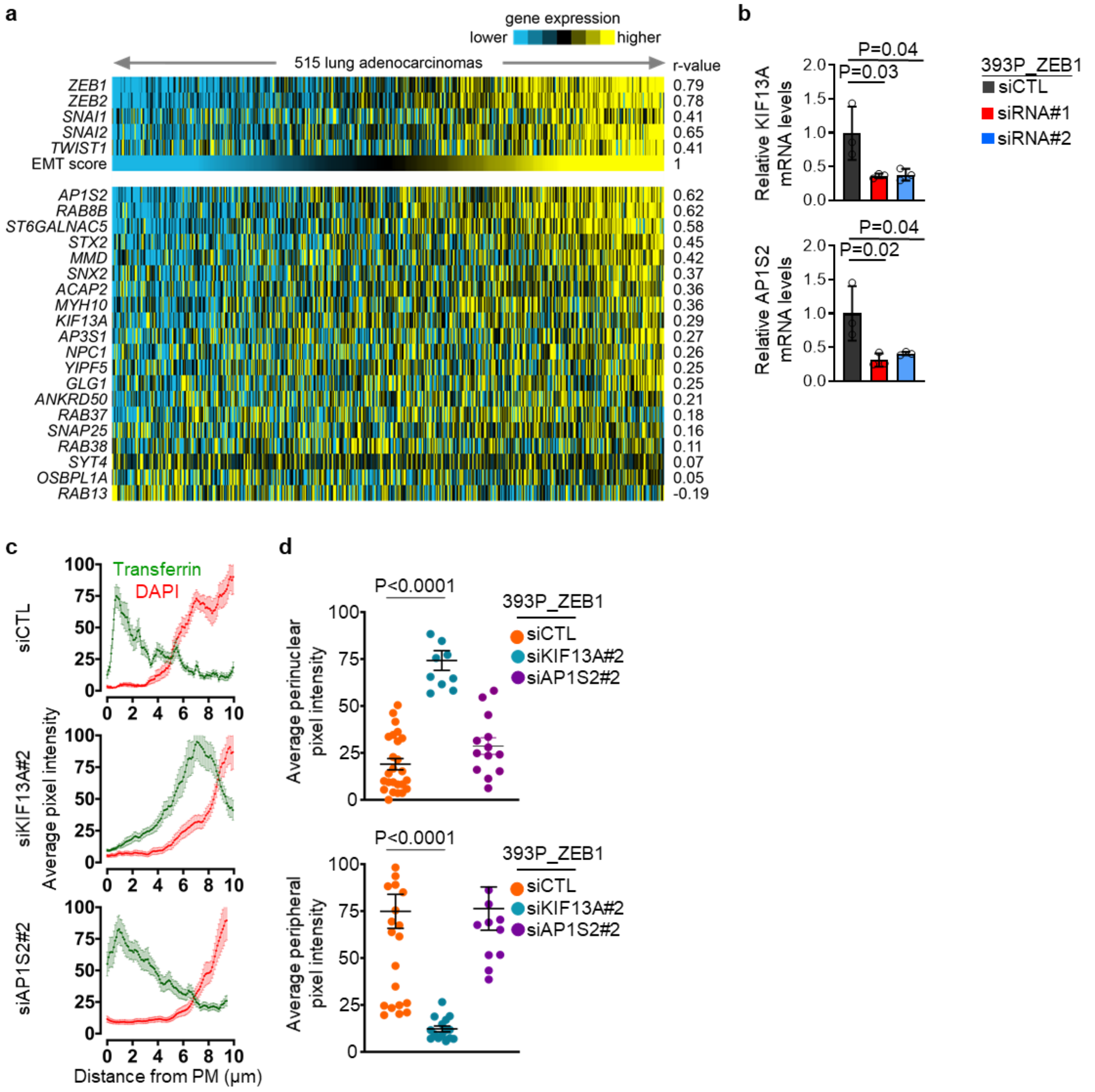

Supplementary Figure 5. High KIF13A levels are associated with EMT and mediate ZEB1-driven endocytic vesicular recycling. (**a**) Heat map depicts correlation between EMT scores and expression levels (high, yellow; blue, low) of vesicular trafficking regulators (rows) in TCGA LUADs (columns). Tumors are ordered from left-to-right based on increasing EMT score. EMT score is based on the Byers *et al*. signature[1] as previously computed across TCGA RNA-seq profiles[2]. Vesicular trafficking regulators (n=20 genes) in the bottom heat map are more highly expressed in 393P_ZEB1 cells than 393P_vector cells[3]. EMT-activating transcription factors (top panel, n=5 genes) included as a control. Gene correlations by Pearson's using log-transformed expression values. Expression values normalized to standard deviations (SDs) from the sample median. (**b**) qPCR analysis of siRNA targets in 393P_ZEB1 cells transfected with KIF13A, AP1S2, or control

(siCTL) siRNAs. n = 3 independent experiments. (**c**) Alexa 568-labelled Tfn and DAPI signal intensities (Y axis) on lines drawn from the PM inwards (X axis) in siRNA-transfected 393P_ZEB1 cells. Cells fixed 10 min after initiating endocytosis. Results represent averages of 3 linescans per cell. n = 25 cells (siCTL), 15 cells (siKIF13A #2), or 12 cells (siAP1S2 #2). (**d**) Tfn signal intensities in perinuclear (top dot plot) and peripheral (bottom dot plot) compartments of each cell (dot). Values represent the maximal signal intensities in each cell. n = 25 cells (siCTL), 15 cells (siKIF13A #2), or 12 cells (siAP1S2 #2). Data are presented as mean values +/- SEM, or as mean values +/- SD (b); R value, Spearman; *P* values, two-tailed Student's t-test.

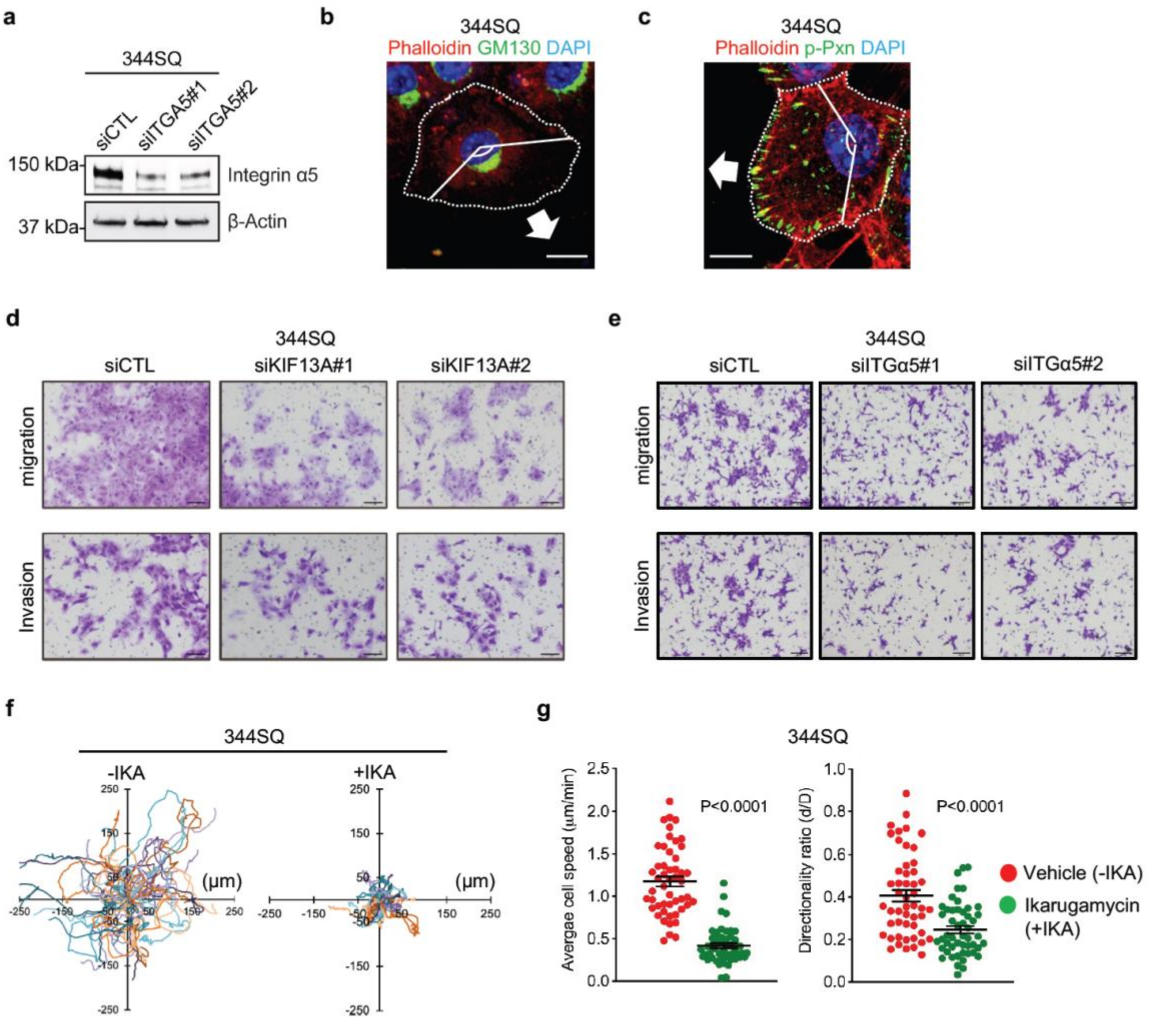

Supplementary Figure 6. ZEB1-dependent endocytic recycling facilitates the establishment of a front-rear polarity axis. (**a**) Western blot analysis of ITGα5 levels in siRNA-transfected 344SQ cells. (**b, c**) Merged confocal micrographs of 344SQ cells with polarized Golgi organelles (**b**) or FAs (**c**) based on staining with anti-GM130 or -phospho-paxillin (p-Pxn) antibodies, respectively. Direction of cell migration on advancing front (arrow). Lines drawn from the center of the nucleus to the lateral edges of the cell describe a 120° arc that defines the polarity quadrant. Scale bars: 20 µm (b), 10 µm (c). (**d, e**) Micrographs of migrated and invaded cells in Boyden chambers. 344SQ cells transfected with KIF13A (**d**) or ITGα5 (**e**) siRNAs. Scale bars: 200 µm. (**f**) Origin-normalized plots of single cell trajectories. 344SQ cells were pre-treated for 3 h with (+) or without (-) 4 µM Ikarugamycin (IKA) and then subjected to 8 h of tracking. (**g**) Speed and directionality were determined for each cell (dot) in (**f**). n = 50 cells from 3 independent experiments. Data are presented as mean values +/- SEM; *P* values, two-tailed Student's t-test.

Supplementary Table 1. List of qPCR primers.

Gene	Forward (5'-3')	Reverse (5'-3')
AP1S2	GATGTGCAGCTTCCTTGAGTGG	TCCACGTAACGATGGATTATTTCC
AP1S2#2	GAAAGCTTCGACTGCAGAAATG	TCTTGGGTTTCCGTGCTAAA
AP3S1	CGCAACAGCAAATCATCAGGGAG	TGAGCTTGTTGTCAGAGCCTCC
GLG1	AAGTGGTGCAGTGAGAAGACGG	CGGACTCTAACTCGGTGAGGTT
ITGα5	ACCTGGACCAAGACGGCTACAA	CTGGGAAGGTTTAGTGCTCAGTC
KIF13A	GACCAGTGTGAGTCTACAGTGG	CCAGGAACTCTTCTGTGACAGTC
KIF13A#2	TGGCTTCGATGAAGACGATAAG	AGGTGAGTCCCTGGGTAAA
Rab13	ATCCGAACCGTGGACATAGAGG	ATGGCTCCACGGTAATAGGCGG
SNX2	TGAGGATGGTGAACAAGGCTGC	AGACCAAGGCTTCAACACTGGC
Tfrc	GAAGTCCAGTGTGGGAACAGGT	CAACCACTCAGTGGCACCAACA
Yipf5	GAAGTGGAGGACCCTACAGCAA	TGCCCAGTGTATGTCTGCTGTG
ZEB1	GCTCAGCCAGGAACCCGCAG	TGGGCACCCTCTGCCACACA

Supplementary References

1. Byers LA*, et al.* An epithelial-mesenchymal transition gene signature predicts resistance to EGFR and PI3K inhibitors and identifies Axl as a therapeutic target for overcoming EGFR inhibitor resistance. *Clin Cancer Res* **19**, 279-290 (2013).

2. Gibbons DL, Creighton CJ. Pan-cancer survey of epithelial-mesenchymal transition markers across the Cancer Genome Atlas. *Dev Dyn* **247**, 555-564 (2018).

3. Yang Y*, et al.* ZEB1 sensitizes lung adenocarcinoma to metastasis suppression by PI3K antagonism. *J Clin Invest* **124**, 2696-2708 (2014).
