# Supplementary material for: The EMT activator ZEB1 accelerates endosomal trafficking to establish a polarity axis in lung adenocarcinoma cells
Source: Nat Commun. 2021 Nov 3;12:6354. doi: 10.1038/s41467-021-26677-y (PMC8566461; doi:10.1038/s41467-021-26677-y)
Supplement: Supplementary file 3 — Description of Additional Supplementary Files [file 41467_2021_26677_MOESM3_ESM.pdf]

Description of Additional Supplementary Files

File Name: Supplementary Movie 1
Description: ZEB1 is a driver of directional endosome motility. Spinning disk confocal time-lapse live-. cell imaging of 393P_Vector cells (left) and 393P_ZEB1 cells (right) stably expressing Rab5-mCherry. Tubular structures (arrows) in 393P_ZEB1 cells are intermediates from early endosomes and represent enhanced maturation. Images were acquired at 1s intervals for 10 min and movie was compiled at 60 frames per second. Scale, 10 µm.

File Name: Supplementary Movie 2
Description: ZEB1 drives endosomal maturation. Confocal time-lapse live-cell imaging of 393P_Vector cells (left) and 393P_ZEB1 cells (right) pulsed with 25 µg/ml pHrodo Red Tfn (top panels). Cell boundaries are visible in transmitted light channel (bottom panels). Progressive appearance of bright centrally located intracellular patches of the dye (red arrowhead) is detectable in 393P_ZEB1 cells but not in 393P_Vector cells. Images were acquired for 20 min at 1-min intervals and a movie was compiled at 4 frames per sec. Scale, 15 µm.

File Name: Supplementary Movie 3
Description: Retrograde vesicular trafficking is faster in mesenchymal than epithelial human LUAD cell lines. Confocal time-lapse live-cell imaging of 393P cells (left) and 344SQ cells (right) treated overnight with BacMam Golgi-RFP (pseudocolored red), pulsed with CTxB (pseudocolored green), and imaged after 1 h. CTxB accumulated in the Golgi in 344SQ cells but not 393P cells. Images were acquired at 30-sec intervals for 60 min, and a movie was compiled at 12 frames per second. Scale, 3 µm.
