# Supplementary material for: The EMT activator ZEB1 accelerates endosomal trafficking to establish a polarity axis in lung adenocarcinoma cells
Source: Nat Commun. 2021 Nov 3;12:6354. doi: 10.1038/s41467-021-26677-y (PMC8566461; doi:10.1038/s41467-021-26677-y)
Supplement: Supplementary file 7 — Reporting Summary [file 41467_2021_26677_MOESM7_ESM.pdf]

nature portfolio

Corresponding author(s): Jonathan M. Kurie (NCOMMS-21-00292B)

Last updated by author(s): 09.04.2021

Reporting Summary

Nature Portfolio wishes to improve the reproducibility of the work that we publish. This form provides structure for consistency and transparency in reporting. For further information on Nature Portfolio policies, see our Editorial Policies and the Editorial Policy Checklist.

Statistics

For all statistical analyses, confirm that the following items are present in the figure legend, table legend, main text, or Methods section.

n/a	Confirmed	
☐	☒	The exact sample size (*n*) for each experimental group/condition, given as a discrete number and unit of measurement
☐	☒	A statement on whether measurements were taken from distinct samples or whether the same sample was measured repeatedly
☐	☒	The statistical test(s) used AND whether they are one- or two-sided<br>*Only common tests should be described solely by name; describe more complex techniques in the Methods section.*
☐	☒	A description of all covariates tested
☐	☒	A description of any assumptions or corrections, such as tests of normality and adjustment for multiple comparisons
☐	☒	A full description of the statistical parameters including central tendency (e.g. means) or other basic estimates (e.g. regression coefficient) AND variation (e.g. standard deviation) or associated estimates of uncertainty (e.g. confidence intervals)
☐	☒	For null hypothesis testing, the test statistic (e.g. *F*, *t*, *r*) with confidence intervals, effect sizes, degrees of freedom and *P* value noted<br>*Give P values as exact values whenever suitable.*
☒	☐	For Bayesian analysis, information on the choice of priors and Markov chain Monte Carlo settings
☒	☐	For hierarchical and complex designs, identification of the appropriate level for tests and full reporting of outcomes
☐	☒	Estimates of effect sizes (e.g. Cohen's *d*, Pearson's *r*), indicating how they were calculated

Our web collection on statistics for biologists contains articles on many of the points above.

Software and code

Policy information about availability of computer code

Data collection	Quantitative RT-PCR data were acquired using ABI 7500 system; Brightfield and high resolution widefield images were collected using Nikon NIS Elements V4.40. ELISA readings were made using SoftMax Pro v6. Confocal images were acquired using Nikon NIS Elements V4.60. Spinning disc confocal images were acquired using Andor iQ V3.
Data analysis	Graph Pad Prism 9 and Microsoft Excel were used to generate graphs and perform statistical analysis. Heat maps were generated using JavaTreeView 1.2.0. Micrographs were processed and analyzed using ImageJ / Fiji 1.53, SVI Huygens Provessional V17.10, and Bitplane Imaris 9.6.

For manuscripts utilizing custom algorithms or software that are central to the research but not yet described in published literature, software must be made available to editors and reviewers. We strongly encourage code deposition in a community repository (e.g. GitHub). See the Nature Portfolio guidelines for submitting code & software for further information.

Data

Policy information about availability of data

All manuscripts must include a data availability statement. This statement should provide the following information, where applicable:
- Accession codes, unique identifiers, or web links for publicly available datasets
- A description of any restrictions on data availability
- For clinical datasets or third party data, please ensure that the statement adheres to our policy

All data associated with this study are present in the paper and the Supplementary Information. Source data are provided with this paper. RNA-seq data that support the findings of this study has been deposited in Gene Expression Omnibus (accession number GSE102337).

Field-specific reporting

Please select the one below that is the best fit for your research. If you are not sure, read the appropriate sections before making your selection.

- [x] Life sciences
- [ ] Behavioural & social sciences
- [ ] Ecological, evolutionary & environmental sciences

For a reference copy of the document with all sections, see nature.com/documents/nr-reporting-summary-flat.pdf

Life sciences study design

All studies must disclose on these points even when the disclosure is negative.

Sample size	No sample size calculation was performed. Data were collected randomly and sample sizes were chosen based on previously published reports. Such sample sizes has been convincingly used to describe similar mechanisms.
Data exclusions	No data were excluded.
Replication	All attempts at replication were successful. Transferrin experiments were validated by blinding the investigators. All experiments were independently repeated at least two times.
Randomization	Cells were randomly seeded into control or experimental groups. Micrographs were acquired randomly across the entire sample.
Blinding	The investigators were blinded to group allocation during data collection and analysis of intracellular transferrin distribution patterns.

Reporting for specific materials, systems and methods

We require information from authors about some types of materials, experimental systems and methods used in many studies. Here, indicate whether each material, system or method listed is relevant to your study. If you are not sure if a list item applies to your research, read the appropriate section before selecting a response.

Materials & experimental systems

n/a	Involved in the study	
[ ]	[x]	Antibodies
[ ]	[x]	Eukaryotic cell lines
[x]	[ ]	Palaeontology and archaeology
[x]	[ ]	Animals and other organisms
[x]	[ ]	Human research participants
[x]	[ ]	Clinical data
[x]	[ ]	Dual use research of concern

Methods

n/a	Involved in the study	
[x]	[ ]	ChIP-seq
[x]	[ ]	Flow cytometry
[x]	[ ]	MRI-based neuroimaging

Antibodies

Antibodies used	We purchased Alexa Fluor tagged secondary antibodies (#A-11055, #A-32787 and #A-10042), and Zenon Mouse IgG2a Labeling Kit (#Z25102) from Thermo Fisher Scientific; primary antibodies against LAMP1 (#9091), MET (#8198), phospho-MET (#3077), phospho-Paxillin (Tyr118) (#2541), Rab11 (#5589) from Cell Signaling Technologies; primary antibody against MET (#AF276), recombinant human HGF protein (#294-HG-005/CF) from R&D Systems. Horseradish peroxidase (HRP)-conjugated secondary antibodies (#7074P2 and #7076P2) were from Cell Signaling Technologies.
Validation	Where applicable, Western blotting was used to determine an antibody's specificity based on: the observation of a single band at the known molecular weight for the target; the absence of that band in negative controls (e.g., no primary antibody, lysates from a cell line or tissue known not to express the protein of interest and/or lysates from a cell line treated with RNAi targeting the gene or interest); and the presence of that band in positive controls (e.g., non-expressing cells transfected/transduced to express the protein of interest).

Eukaryotic cell lines

Policy information about cell lines

Cell line source(s)	Described in the "Reagents" subsection of Methods section.
Authentication	Human cell lines were obtained from a validated source (e.g., ATCC; http://www.atcc.org/) that provides evidence to guarantee authenticity and absence of microbial contamination including mycoplasma. We verified that the cell lines utilized are not on the International Cell Line Authentication Committee (ICLAC) list of misidentified cell lines (http://iclac.org/databases/cross-contaminations/). We confirmed freedom from mycoplasma and other microorganisms. Murine lung cancer cell lines are authenticated by PCR-based confirmation of the presence of mutant alleles (KrasG12D and Tp53R172H) and the

	ectopic expression or depletion of genes in derivatives of the parental cell lines.
Mycoplasma contamination	Cell lines were documented to be free of mycoplasma.
Commonly misidentified lines (See ICLAC register)	No commonly misidentified lines were used.
